# Identification of super-enhancer-driven peptidyl arginine deiminases as potential biomarkers and therapeutic targets for osimertinib-resistant non-small cell lung cancer

**DOI:** 10.3389/fphar.2022.1071365

**Published:** 2022-11-21

**Authors:** Hang Li, Gulizeba Muhetaer, Yizi Xie, Kainan Yao, Qianqian Ma, Huiting Guan, Sizhong Xing, Xiufang Huang, Jihong Zhou

**Affiliations:** ^1^ Shenzhen Bao’an Traditional Chinese Medicine Hospital, Guangzhou University of Chinese Medicine, Shenzhen, China; ^2^ The First Affiliated Hospital of Guangzhou University of Chinese Medicine, Guangzhou, China; ^3^ Lingnan Medical Research Center of Guangzhou University of Chinese Medicine, Guangzhou, China

**Keywords:** drug resistance, osimertinib, peptidyl arginine deiminase, super-enhancer, non-small cell lung cancer

## Abstract

Resistance to targeted drugs is now a challenging clinical problem in the treatment of non-small cell lung cancer (NSCLC). So far, there are no approved targeted therapeutic drugs for patients with disease progression after the third-generation epidermal growth factor receptor-tyrosine kinase inhibitor osimertinib resistance (OR). Super-enhancers (SEs) are large clusters of transcriptional enhancers that drive gene expression. In this study, we aimed to explore the potential pathogenic SEs and their driven genes in OR NSCLC. OR cell line was established by exposure of H1975 cells to incremental dosing of osimertinib. RNA-sequencing and H3K27ac ChIP-sequencing were used to identify the differential expressed genes (DEGs) and SEs in parental and resistant cells. Gene ontology analysis for the OR-specific SEs-associated genes showed that histone citrullination, protein citrullination, and peptidyl-arginine modification are the top three biological processes, and the DEGs involved in these biological processes, including peptidyl arginine deiminase 1 (PADI1), PADI2, and PADI3. Realtime-PCR and western blot detections confirmed these genes were highly expressed in OR cells. SE inhibitor decreases their expression, ensuring that SEs regulate their transcriptional expressions. The PADI inhibitor inhibited OR cells’ proliferation, invasion, and colony formation. This study demonstrates that SE-driven PADI family genes are potential biomarkers and targets for OR NSCLC.

## Introduction

Lung cancer is the first cancer in men and the second in women worldwide (Sung et al., 2021). Non-small cell lung cancer (NSCLC) accounts for about 85% of lung cancers. Targeted drugs have opened a new situation for the treatment of NSCLC. Epidermal growth factor receptor-tyrosine kinase inhibitors (EGFR-TKIs) are targeted drugs for EGFR mutations. However, resistance to the first- and second-generation EGFR-TKIs is common, and T790M mutation is the most common resistance mechanism (Graham et al., 2018). The third-generation EGFR-TKI osimertinib (OSI) is the first-line treatment for patients with EGFR driver gene-positive advanced NSCLC and a second-line drug for T790M mutation. However, after the first-line or second-line use of OSI, resistance is still inevitable (Mok et al., 2017; Soria et al., 2018), which limits its long-term clinical benefit. So far, there are no approved treatments for patients who develop disease progression after third-generation EGFR-TKI therapy. The mechanism of OSI resistance (OR) is not very clear. Clarifying the mechanism of OR and overcoming its resistance is an important issue that needs to be solved.

An enhancer is a conserved short segment of DNA, generally several hundred base pairs in length. As a key regulatory hub for integrating environmental cues and encoding genome expression, it is essential to control various biological processes in mammalian development (He et al., 2019). In 2013, Professor Young R.A. first discovered some large clusters of transcriptional enhancers essential for maintaining the stemness of embryonic stem cells and defined them as super-enhancers (SEs) (Whyte et al., 2013). SEs can drive the expression of cell identity-related genes (Hnisz et al., 2013). They are closely related to the expression of pathogenic genes in many diseases, including tumorigenesis, Alzheimer’s disease, diabetes, and autoimmune diseases (Lovén et al., 2013; Shin, 2018). Studies have found that SEs mark lineage-specific transcription factors and oncogenes in many tumors (Sengupta and George, 2017). SEs play a role in maintaining tumor characteristics and are associated with higher tumor clinical stages and pathological grades (Han et al., 2019).

Some studies have already reported SEs are associated with drug resistance to the tumor, making them potential therapeutic targets for this challenge. Bao et al. discovered multiple chemoresistance-associated SEs in NSCLC and identified the downstream genes of these SEs associated with doxorubicin, cisplatin, and etoposide resistance, including interferon regulatory factor 1 and Specific protein 1, etc. (Bao et al., 2019). The SOX9 transcription factor regulated by SE contributes to chemoresistance in ovarian cancer (Shang et al., 2019). Early inhibition of the expression of SEs-related genes c-FLIP and XIAP by bromodomain and extraterminal domain (BET) inhibitors can effectively overcome the resistance of NSCLC cells to pro-apoptotic drugs (Klingbeil et al., 2016). Targeting and intervening transcription factors of SEs can significantly overcome the resistance of breast cancer to AKT inhibitors *in vivo* and *in vitro* (Liu et al., 2018).

This study will explore the potential pathogenic SEs and their driven genes in OSI-resistant NSCLC. It will help research the drug resistance mechanism and develop new therapeutic methods for drug-resistant NSCLC.

## Materials and methods

### Cell culture

**FIGURE 1:**
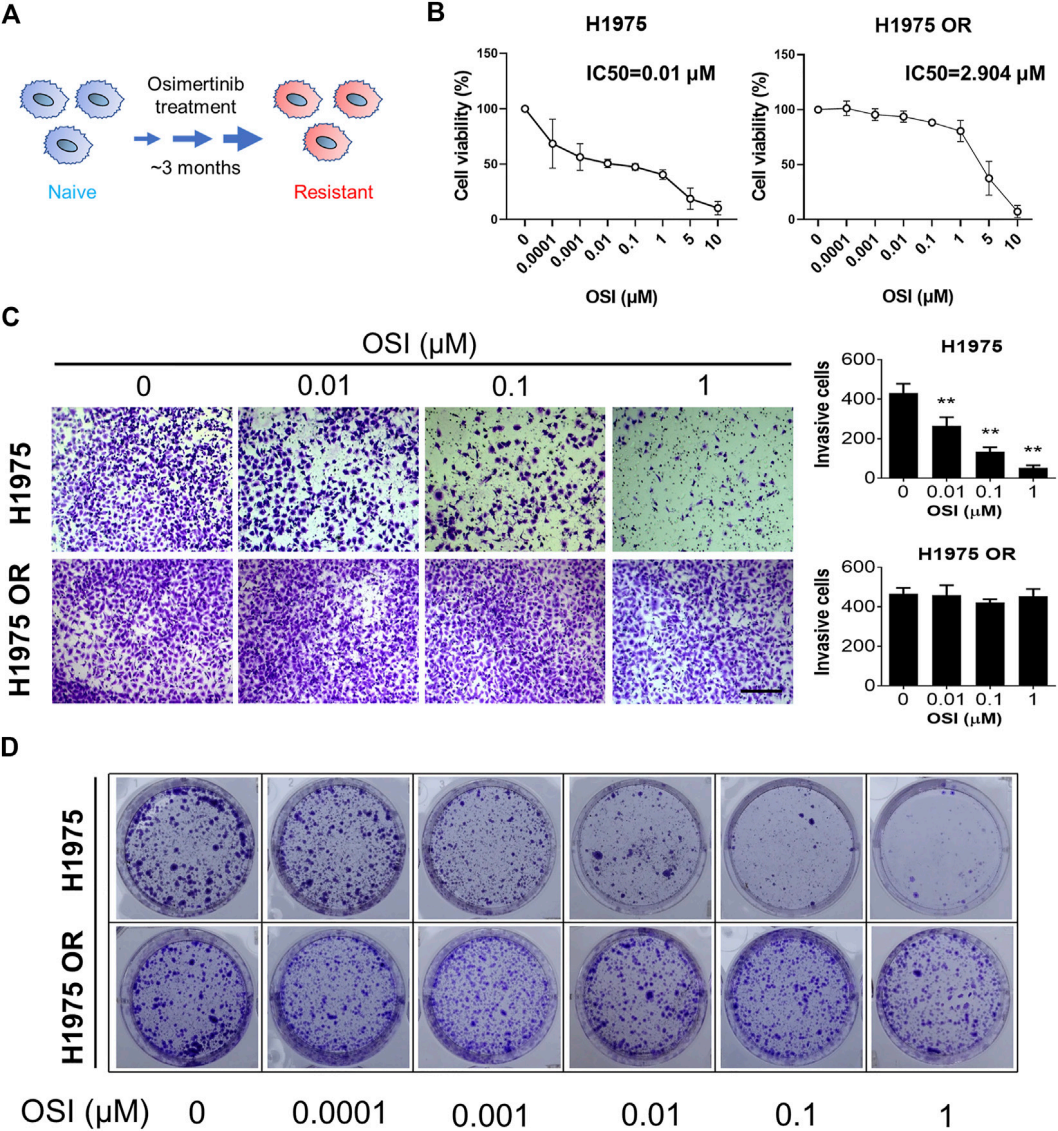
OSI-resistant NSCLC cells were established. **(A)** A schematic diagram shows OSI-resistant cell line was established by continuous exposure of H1975 cells to incremental dosing of OSI for more than 3 months **(B)** The graph shows the percent cell viability (CCK-8 assay) of naive and resistant H1975 cells after 72-h treatment with the indicated doses of OSI. **(C)** Transwell invasion assays for naive and resistant H1975 cells upon exposure to the indicated doses of OSI for 12 h. Column graphs show the quantitative measurement of invasive H1975 cells and H1975 OR cells. The length of the scale bar is 400 μm. **(D)** Colony formation assay shows the inhibitory effect of indicated doses of OSI on colony formation of H1975 cells and H1975 OR cells. Data are presented as mean ± SD. (**p < 0.01 vs. OSI 0 μM; *n* = 3–5).

NCI-H1975 (H1975) cells were purchased from the National Collection of Authenticated Cell Cultures (Shanghai, China). Cells were cultured in a RPMI 1640 medium (Gibco, United States) supplemented with 10% fetal bovine serum (Sigma, United States) and 1% Penicillin-Streptomycin (Gibco, United States). Cells were grown in a 5% CO_2_ incubator at 37°C. Similar to previous reports (Wen et al., 2020), an OSI-resistant cell line (H1975 OR) was established by continuous exposure of H1975 cells to incremental dosing of OSI from 0.01 nM to 1.5 μM for more than 3 months (Figure 1A).

### CCK-8 assay

Cell viability was measured using a Cell Counting Kit-8 (CCK-8) kit (Biosharp, China). Cells were seeded into the 96-well plate (5×10^3^ cells/per well). After cell attachment, cells were treated with different concentrations of OSI or other compounds for 72 h. After that, the original medium was removed, and a new culture medium with 10% CCK-8 was added to each well. The cells were incubated for 2.5 h. Then the absorbance of each well was measured at 450 nm using a multifunctional microplate reader (Bio-Tek, United States).

### Transwell assay

Cell invasion assay was conducted using Matrigel Invasion Chambers (Corning, New York, NY, United States). The upper transwell chambers were seeded with H1975 or H1975 OR cells (1×10^4^ cells in 300 μl serum-free medium). The lower transwell chambers were then filled with 500 μl of RPMI 1640 containing 10% fetal bovine serum. After 12 h, 4% paraformaldehyde was applied to the invaded cells on the lower membrane surface for 10 min. After that, the cells were stained with 1% crystal violet solution (Solarbio, China) for 20 min.

### Colony formation assay

A colony formation assay was performed to investigate further the cyclic inhibition of H1975 or H1975 OR cell growth before and after the OSI administration intervention. In brief, H1975 or H1975 OR cells were seeded in 6-well plates (3×10^3^ cells/per well). After cell attachment, cells were treated with OSI or Cl-amidine for 6 hours, and then the culture was continued for 2 weeks. The resulting colonies were fixed using 4% paraformaldehyde and stained with 1% crystal violet.

### RNA-sequencing

RNA from cells was extracted using TRIzol (Life Technologies, California, United States). For the naïve and OSI-resistant cells experiment, the RNA samples were sent to BGI Tech Company (Shenzhen, China) to perform RNA-seq using the BGISEQ platform following the standard procedures. The raw data were deposited in the Sequence Read Archive (SRA) database (Accession number: PRJNA834908). The differential expressed genes (DEGs) were defined as |log2FC| ≥ 2 and Q value ≤ 0.05. For the JQ1-treated and -untreated H1975 OR cells experiment, the RNA samples were sent to Wekemo Tech Company (Shenzhen, China) to perform RNA-seq using the novaseq-PE150 platform following the standard procedures. The DEGs were defined as |log2FC| ≥ 1 and Q value ≤ 0.05. The raw data were deposited in the SRA database (Accession number: PRJNA853904).

### Chromatin immunoprecipitation-sequencing

ChIP assay was conducted in H1975 and H1975 OR cells using the SimpleChIP Plus Enzymatic Chromatin IP Kit (Magnetic Beads) (Cell Signaling Technology, United States). Cells were lysed, and chromatin was harvested and fragmented (150–900 base pairs) using sonication and enzymatic digestion. The chromatin was subjected to immunoprecipitation using either anti-Histone H3 lysine 27 site acetylation (H3K27ac) (ChIP Grade, ab4729, Abcam) or anti-IgG antibody. After immunoprecipitation, the protein-DNA cross-links were relieved, and the DNA was purified. Then the DNA samples were sent to BGI Tech Company (Shenzhen, China) to perform ChIP-sequencing (ChIP-seq). The raw data were deposited in the SRA database (Accession number: PRJNA834760).

### SE analysis

The ROSE (Rank Ordering of Super-Enhancers) algorithm developed by the team of Richard A. Young (Whyte et al., 2013) was used to identify SEs in the genome of H1975 and H1975 OR cells based on the H3K27ac ChIP-seq data. The analysis procedure is like previously reported (Si et al., 2021). Enhancers were identified through the mapped H3K27ac peak. Enhancers within 12.5 kb of one another were stitched together. Enhancers above the inflection point of the ranking curve (slope >1) were defined as SEs. The ROSE Genemapper algorithm was used to annotate the associated genes of SEs. The H3K27ac ChIP-Seq binding profiles were visualized using the Integrative Genomics Viewer software (version: 2.10.3).

### Gene enrichment analysis

The overlapping genes identified in OR-specific SEs and 10-fold up-regulated genes in H1975 OR cells were defined as SE-associated genes in OR cells. The gene list was uploaded to DAVID Bioinformatics Resources (Huang da et al., 2009) (https://david.ncifcrf.gov/home.jsp) to perform the gene ontology (GO) analysis. Involved biological processes (BP) were obtained.

### Western blotting

Cells were lysed using RIPA buffer (Sigma, United States) with 1% Halt Protease& Phosphatase Inhibitors Cocktail (Thermo Scientific, United States). The concentration of protein solutions was quantified using the Pierce BCA protein assay kit (Thermo Scientific, United States). Protein was separated by NewFlash Protein AnyKD PAGE (Dakewe, Shenzhen, China) and transferred to 0.2 μm PVDF membranes (Millipore, Ireland). After blocking with QuickBlock Blocking Buffer for Western Blot (Beyotime, China). Membranes were incubated with primary antibodies, including peptidyl arginine deiminase 1 (PADI1, Abcam, Cambridge, MA, United States), PADI2 (Proteintech, Wuhan, China), PADI3 (Abcam, Cambridge, MA, United States), and *β*-Tubulin (Proteintech, Wuhan, China) at 4°C overnight, and then incubated with the corresponding secondary antibody for 1 h at room temperature. Protein bands were visualized using a chemiluminescent HRP substrate (Millipore, United States).

### Total RNA extraction and RT-qPCR

Total RNA from cells was isolated using the RNA Easy Fast Tissue/Cell Kit (Tiangen, China). After that, RNA was converted to cDNA using All-in-One First-Strand cDNA Synthesis SuperMix for qPCR (Transgen, Beijing, China). Real-time PCR was performed using the TB Green™ Premix Ex Taq™ II Kit (Takara, Tokyo, Japan) on an LC480 real-time PCR system (Roche, Basel, Switzerland). Experiments were performed in triplicate. All data were quantified using the 2^−ΔΔCT^ method in relative quantification and normalized to GAPDH mRNA expression. The primer sequences of the target genes are shown in Table 1.

**TABLE 1:** Primer sequences.

Gene	Sequence (5′-3′)
PADI1 forward	CCT​CAC​TGG​CGT​CGA​TAT​TT
PADI1 reverse	TCA​CAG​TTC​ACC​AGC​AAG​ATA​G
PADI2 forward	GGG​AAG​GAG​ATT​CTG​GGA​TTG
PADI2 reverse	GCA​GTT​CAA​GAT​ACG​TCT​AGG​G
PADI3 forward	GGC​CAA​GAT​AAG​GTG​TCC​TAT​G
PADI3 reverse	CAG​AGT​GAC​ATG​GAA​GGA​GAT​G
GAPDH forward	GGT​GTG​AAC​CAT​GAG​AAG​TAT​GA
GAPDH reverse	GAG​TCC​TTC​CAC​GAT​ACC​AAA​G

### Statistical analysis

All data were expressed as the mean ± standard deviation (SD). GraphPad Prism software (version 8.2.1) was used to ascertain statistically significant differences. A two-tailed Student’s t test was employed to explore the difference between the two groups. The differences among multiple groups were evaluated using the one-way analysis of variance or two-way ANOVA. *p* < 0.05 was considered statistically significant.

## Results

### OSI-resistant NSCLC cells were established

OSI-resistant H1975 cells were established from H1975 cells through culture with incremental dosing of OSI. There was a significant inhibition in the cell viability of H1975 cells after 72 h of OSI dosing treatment (Figure 1B). The half-maximal inhibitory concentration (IC50) value of OSI for H1975 was 0.01 μM. Compared to H1975 cells, increased doses of OSI did not show an apparent toxic effect on H1975 OR cells. The IC50 value of OSI for H1975 OR was 2.904 μM (Figure 1B). Furthermore, transwell invasion assays showed a significant decrease in the number of invading cells after OSI dosing treatment in H1975 cells, indicating that OSI diminished the invasion potential of H1975 cells (Figure 1C). However, the number of invasive cells did not change after the same concentrations of OSI were applied to H1975 OR cells. To further verify the resistance of H1975 OR cells to OSI, we examined the colony formation ability of H1975 and H1975 OR cells after OSI dosing treatment. Cell colony formation gradually decreased when H1975 cells were treated with 0.01–1 μM of OSI (Figure 1D). However, the colony formation of H1975 OR cells did not change significantly under the same concentration of OSI treatment, even at 1 μM OSI concentration (Figure 1D).

### RNA-seq identified the DEGs in OSI-resistant NSCLC cells

**FIGURE 2:**
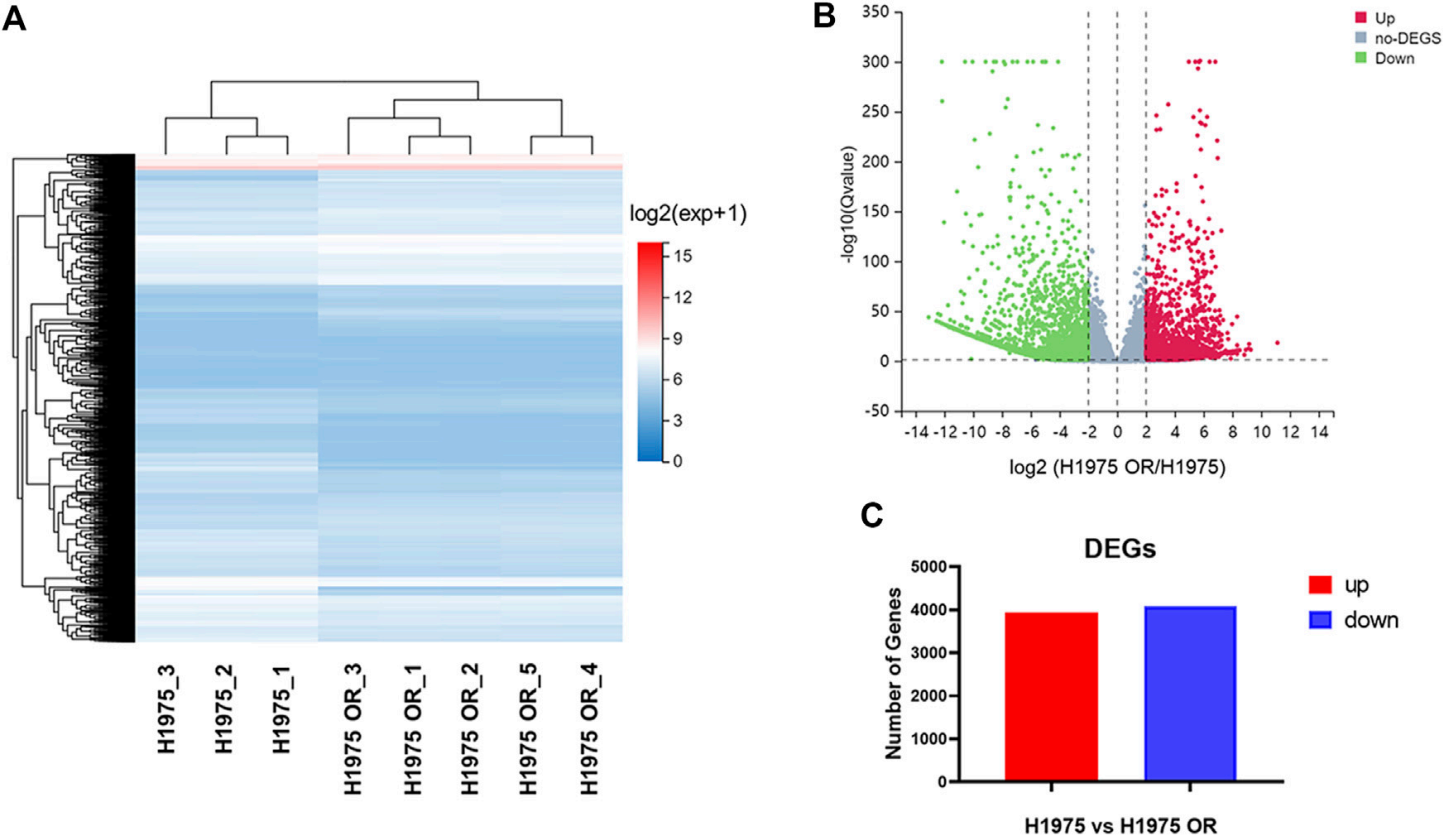
RNA-seq identified the DEGs in OSI-resistant NSCLC cells. **(A)** The cluster heatmap shows the expression of DEGs between H1975 cells and H1975 OR cells detected by RNA-seq. **(B)** The volcano plot exhibits the up-regulated DEGs, no-DEGs, and downregulated DEGs between H1975 cells and H1975 OR cells. **(C)** The graph shows the number of up-regulated and down-regulated differentially expressed gene transcripts in H1975 OR cells compared to H1975 cells.

RNA-seq technique was used to detect DEGs between parental and resistant H1975 cells. The cluster heatmap (Figure 2A) and volcano map (Figure 2B) demonstrated the DEGs between the two groups. It was shown that there were 8,022 differential expression transcripts, of which 3,946 were up-regulated, and 4,076 were down-regulated in resistant cells compared with parental cells (Figure 2C).

### OR-specific SEs were identified

**FIGURE 3:**
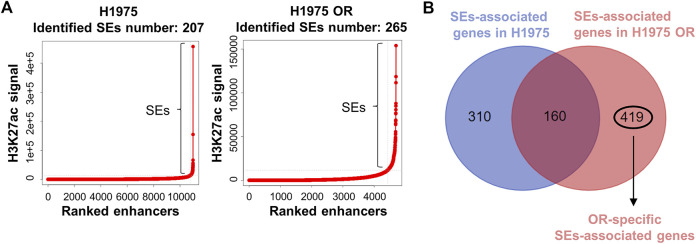
OR-specific SEs were identified. **(A)** Dot plots show H3K27ac read coverage of distal enhancers in indicated cells. A total of 207 SEs and 265 SEs were identified above a flex-point (slope >1) in H1975 cells and H1975 OR cells. **(B)** Venn diagram shows the intersection of SEs-associated genes in H1975 cells and H1975 OR cells. OR-specific SEs-associated genes were marked.

H3K27ac is a commonly used marker for identifying SEs. We performed the H3K27ac Chip-seq technique to determine the chromatin binding of H3K27ac in H1975 cells and H1975 OR cells. Using biological ROSE algorithm analysis, 207 SEs were identified in H1975 cells and 265 SEs in H1975 OR cells (Figure 3A). It is known that SEs drive the expression of close genes, which are called SEs-associated genes. There are 470 SE-associated genes in H1975 cells and 579 SE-associated genes in H1975 OR cells predicted by the genemapper algorithm. By intersecting SEs-associated genes in parental and drug-resistant cells, 419 SEs specific to H1975 OR cells were identified (Figure 3B).

### PADI family genes were OR-specific SEs-associated genes

**FIGURE 4:**
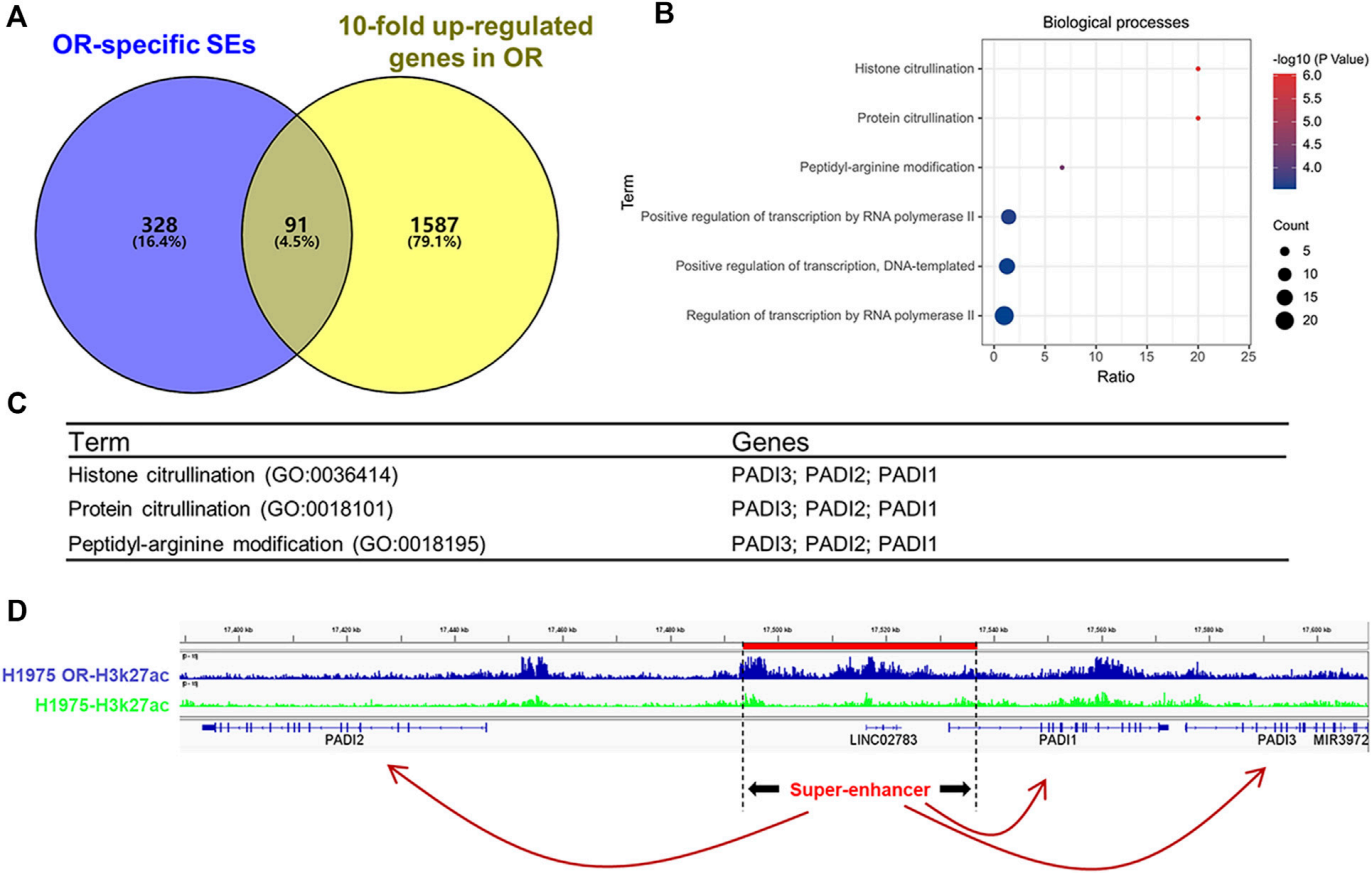
PADI family genes were OR-specific SEs-associated genes. **(A)** Venn diagram shows the intersection of OR-specific SEs-associated genes and genes that are more than 10-fold up-regulated in H1975 OR cells. **(B)** Gene ontology (GO) analysis of enriched biological processes for the 91 OR-specific SEs-associated genes. **(C)** The genes involved in the top 3 enriched biological processes. **(D)** H3K27ac ChIP-Seq binding profiles show the SE and associated PADI genes in H1975 cells and H1975 OR cells.

Considering the superpower of SEs that can drive gene expression several times, even tens of times higher than typical enhancers do. So, we set 10-fold as the parameter to screen SEs-associated genes in up-regulated DEGs of OR cells and got 1,678 genes (Figure 4A). Venn diagram of OR-specific SEs-associated genes and genes that were more than 10-fold up-regulated in H1975 OR cells revealed that 91 genes were intersected (Figure 4A). Gene enrichment analysis for these 91 genes showed that histone citrullination, protein citrullination, and peptidyl-arginine modification were the top 3 BPs (Figure 4B). And the DEGs involved in these biological processes all belonged to the PADI family, including PADI1, PADI2, and PADI3 (Figure 4C). The position of SE driving the PADI family genes (PADI SE) in the genome of H1975 OR cells is chr1:17493428–17536922, marked in red in Figure 4D. We can see that compared to the H1975 cells, more H3K27ac peaks are enriched in PADI SE in H1975 OR cells. PADI1, PADI2, and PADI3 genes are all close to PADI SE.

### PADI1, PADI2, and PADI3 were highly expressed in H1975 OR cells

**FIGURE 5:**
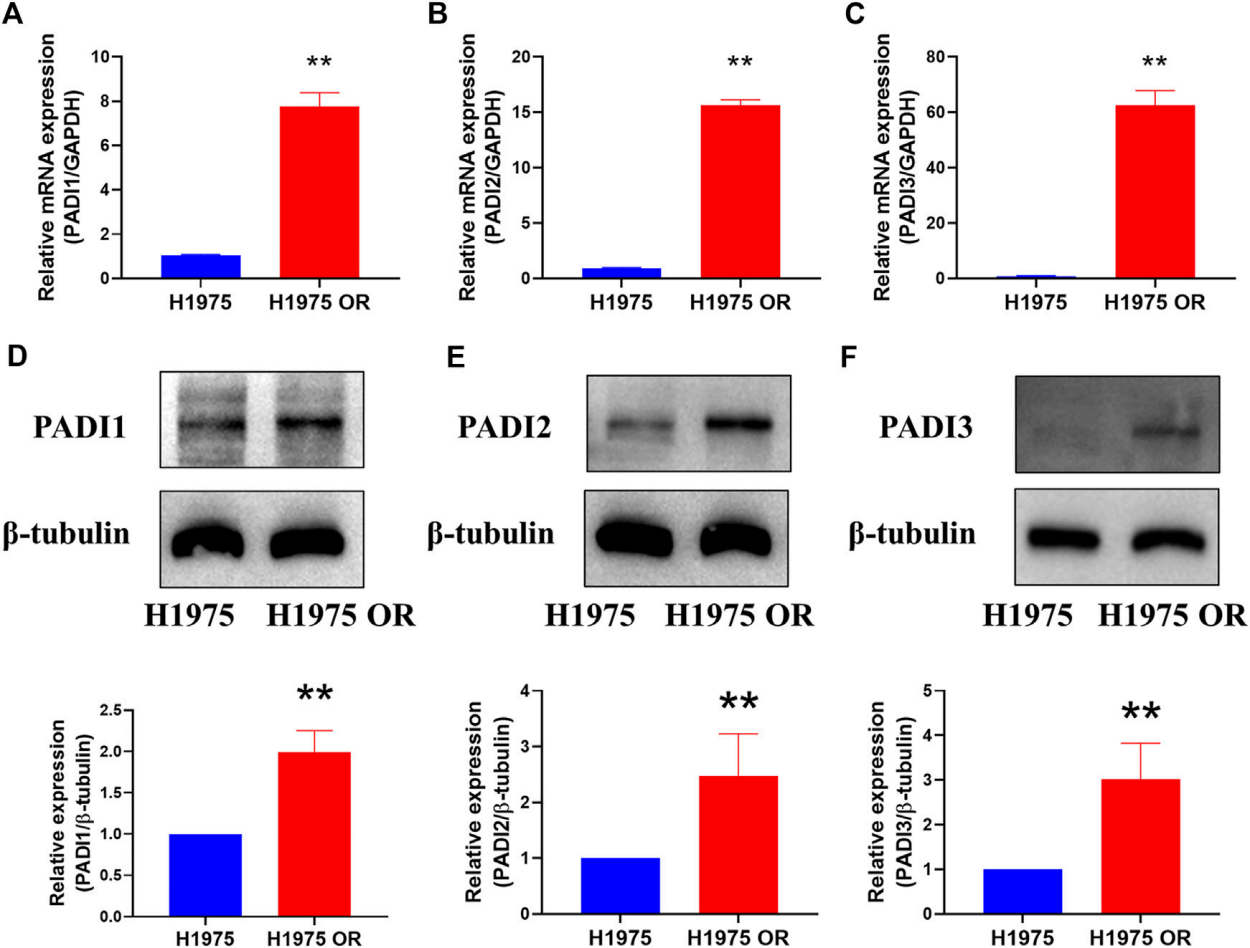
PADI1, PADI2, and PADI3 were highly expressed in H1975 OR cells. **(A–C)** The mRNA expressions of PADI1, PADI2, and PADI3 in H1975 cells and H1975 OR cells were detected by real-time PCR. **(D–F)** Representative bands of western blots show protein expression of PADI1, PADI2, PADI3, and *β*-tubulin in H1975 cells and H1975 OR cells. Data are presented as mean ± SD. (**p < 0.01 vs. H1975; *n* = 3–5).

To verify the high expression of PADI1, PADI2, and PADI3 in OR, we examined the mRNA levels of these genes in naïve and OR cells by real-time PCR. The mRNA expressions of PADI1, PADI2, and PADI3 were significantly increased in H1975 OR cells compared with H1975 cells (Figures 5A–C). At the protein level, similar results were observed (Figures 5D–F). Proteins of PADI1, PADI2, and PADI3 were highly expressed in H1975 OR cells compared to H1975 cells.

### SE inhibitor decreased the expression of PADI1, PADI2, and PADI3 in H1975 OR cells

**FIGURE 6:**
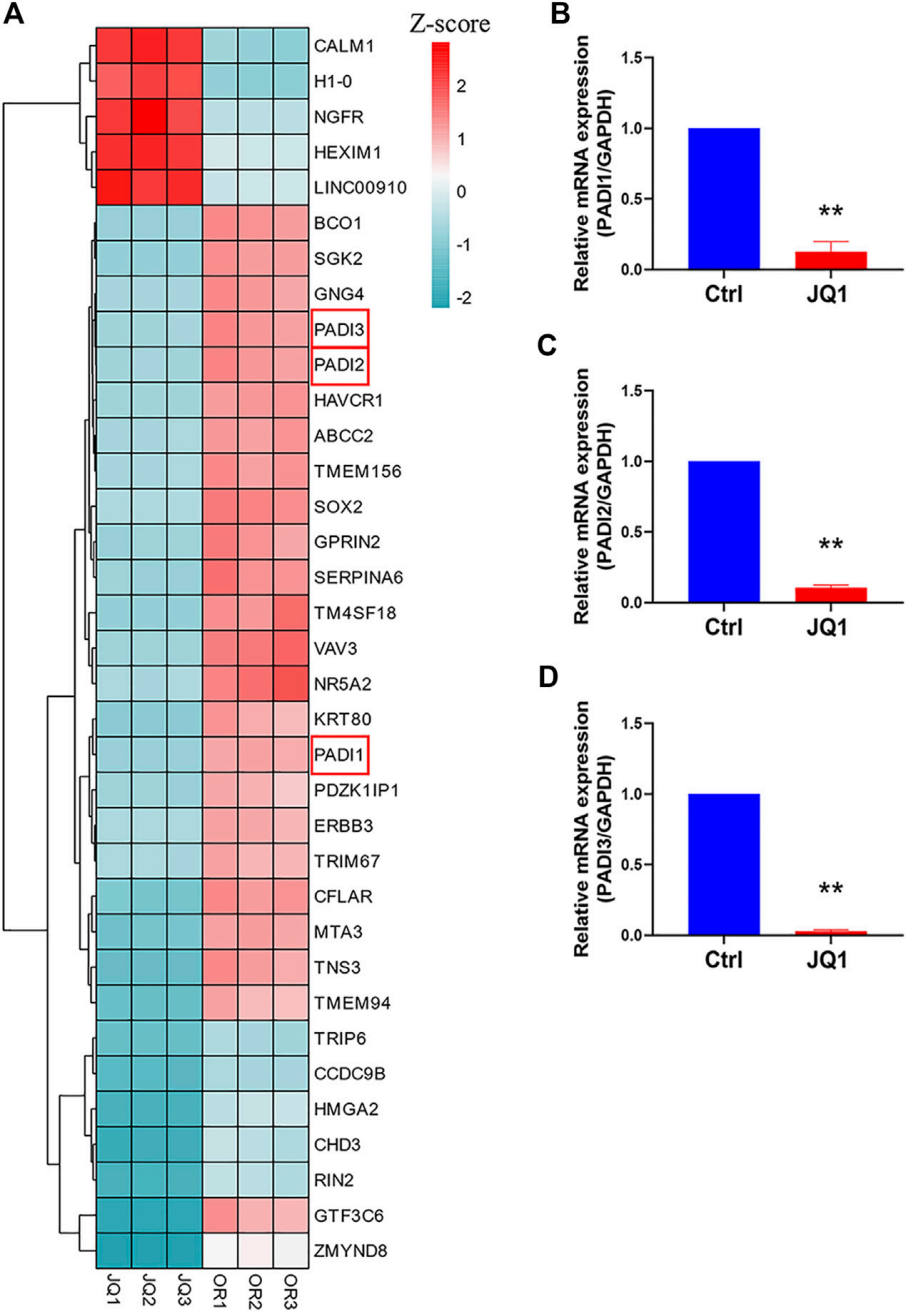
SE inhibitor decreased the expression of PADI1, PADI2, and PADI3 in H1975 OR cells. **(A)** The heatmap graph shows the expression of the top 35 DEGs between JQ1-treated and untreated H1975 OR cells. **(B–D)** The mRNA expressions of PADI1, PADI2, and PADI3 in H1975 OR cells were detected by real-time PCR after 10 µM JQ1 treatment for 12 h. Data are presented as mean ± SD. (**p < 0.01 vs. Ctrl; *n* = 3).

JQ1, a BET inhibitor, preferentially inhibits SE signaling and has been widely studied for treating diseases with disorder SE activities (Chen et al., 2018). In the previous steps, we inferred that PADI family genes were OR-specific SEs-associated genes. But whether SEs drive their transcriptions is not sure. To confirm that, we performed RNA-seq to detect the DEGs after JQ1 was applied to H1975 OR cells. PADI1, PADI2, and PADI3 were in the top 35 DEGs (Figure 6A). Their mRNA expressions were further proved to be inhibited by JQ1 treatment (Figures 6B–D).

### PADI inhibitor inhibited the proliferation, invasion, and colony formation of H1975 OR cells

**FIGURE 7:**
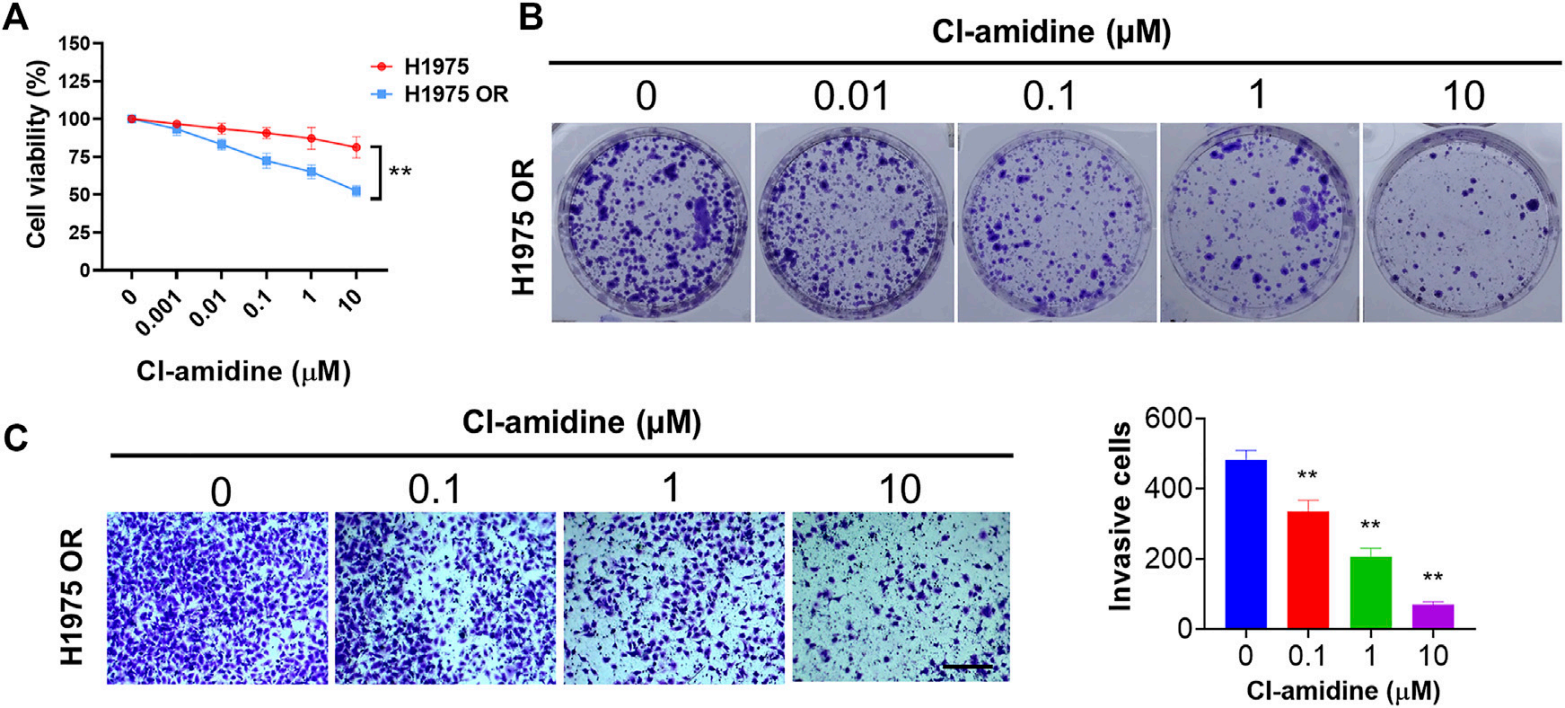
PADI inhibitor inhibited the proliferation, colony formation, and invasion of H1975 OR cells. **(A)** The graph shows the percent cell viability (CCK-8 assay) of H1975 cells and H1975 OR cells after 72-h treatment with the indicated doses of Cl-amidine. Data are presented as mean ± SD. (**p < 0.01 vs. H1975; *n* = 3). **(B)** Colony formation assay shows the inhibitory effect of indicated doses of Cl-amidine on colony formation of H1975 OR cells. **(C)** Transwell invasion assays for H1975 OR cells upon exposure to the indicated doses of Cl-amidine for 12 h. The length of the scale bar is 400 μm. Column graphs show the quantitative measurement of invasive cells. Data are presented as mean ± SD. (**p < 0.01 vs. Cl-amidine 0 μM; *n* = 3).

Cl-amidine is an irreversibly pan-PADI inhibitor. As shown in Figure 7A, Cl-amidine did not demonstrate a significant killing effect on H1975 cells. Interestingly, when applied to H1975 OR cells, Cl-amidine had a significant inhibitory effect on cell viability. Next, we found that Cl-amidine inhibited colony formation of H1975 OR cells (Figure 7B). Furthermore, transwell invasion assays showed a significant decrease in invading cells after Cl-amidine dosing treatment in H1975 OR cells (Figure 7C).

## Discussion

Resistance to targeted drugs is now a challenging clinical problem in treating tumors. EGFR TKIs are the first-line therapeutic drug for EGFR mutations in NSCLC. Unfortunately, drug resistance to the first- (gefitinib and erlotinib) and the second-generation (afatinib) EGFR TKI is very common, with average progression-free survival ranging from 9 to 15 months (Recondo et al., 2018). As a third-generation EGFR TKI highly selective for EGFR-activating mutations, OSI is not only approved for the treatment of T790M-positive patients who have progressed on first- or second-generation EGFR-TKIs, but also as first-line therapy for advanced EGFR-mutated NSCLC, regardless of T790M mutation status (Soria et al., 2018; Yi et al., 2019). However, NSCLC patients inevitably develop resistance to OSI without further better therapeutic options (Leonetti et al., 2019). Resistance to OSI can be divided into on-target EGFR-dependent mechanisms and off-target EGFR-independent mechanisms (Schmid et al., 2020). EGFR-independent mechanisms are mainly related to alternate pathway activation or aberrant downstream signaling. The mechanism of OSI resistance is not completely clear. Figuring out the mechanism will help to find novel therapeutic drugs after OSI resistance. Some studies have shown a close relationship between SEs and drug resistance to tumors (Sava et al., 2020; Wen et al., 2020). This study tried to research the role of pathogenic SEs in acquired OR in NSCLC.

Generally, OSI is used as second-line therapy in patients with T790M positive after the failure of a first-line TKI. Drug resistance occurs after 9 months of OSI as the second-line use or 19 months as the first-line use. H1975 cell is a gefitinib-resistant (GR) NSCLC cell line with EGFR T790M mutation. To simulate the clinical scene where NSCLC patients develop back-to-back GR and OR after taking gefitinib and OSI, we use incremental dosing of OSI to treat H1975 cells. The induced cell line is proven resistant to high OSI doses. High doses (under 1 µM) of OSI had no significant inhibitory effect on cell proliferation, invasion, or colony formation of H1975 OR cells. This cell model ultimately has GR and OR at the same, which is consistent with clinical practice and appropriate for exploring the drug for OR NSCLC.

The transformation from OSI-sensitive cells to OSI-resistant cells could be considered a change in cell identity. SEs play an essential role in the control of cell identity (Hnisz et al., 2013). Some studies have reported that SEs take part in drug-resistance of tumors, such as NSCLC (Bao et al., 2019), ovarian cancer (Shang et al., 2019), and breast cancer (Liu et al., 2018). SEs contribute to chemoresistance by regulating cancer stem cell formation, cellular plasticity, the microenvironment, chemoresistance genes, non-coding RNAs, and tumor immunity (Li et al., 2021). SE-associated long non-coding RNA (SE-lncRNA) is a type of lncRNAs driven by SE. It was reported that SE-lncRNA smooth muscle and endothelial cell-enriched migration/differentiation-associated lncRNA (SENCR) were highly overexpressed in cisplatin-resistant A549/DDP cells, and it promoted cisplatin resistance and growth of NSCLC through upregulating FLI1 (Shen et al., 2022).

In our study, we tried to identify the pro-OR SEs and explore them as potential targets for OR NSCLC. The number 265) of SEs identified in H1975 OR cells is more than that (207) in H1975 cells, indicating that some newly formed SEs may take part in the happening of OR. By identifying SEs and their associated genes in H1975 cells and H1975 OR cells, we found that 419 genes are OR-specific SEs-associated genes. These genes-related SEs are potential SEs result in OR status. Considering the super transcriptional capabilities of SEs, it is necessary to narrow the scope of true pro-OR SEs using the highly expressed DEGs. Therefore, we overlapped OR-specific SEs-associated genes and 10-fold up-regulated DEGs of OR cells and obtained 91 pro-OR SEs-associated genes.

GO analysis for these genes was performed to determine what functions these pro-OR SEs-associated genes may be involved in. The top three enriched BPs are histone citrullination, protein citrullination, peptidyl-arginine modification, and three PADI family genes (PADI1, PADI2, and PADI3) were involved in these BPs. Moreover, the GO analysis of BPs for OR-specific SEs-associated genes also shows that these genes are involved in the positive regulation of transcription. We believe that under the OR state, more SEs are generated, and associated genes are transcribed to resist the toxic effect of targeted drugs and maintain the OR feature. Nucleosomes with the H3K27ac histone modifications are enriched at active enhancers (Creyghton et al., 2010). The levels of H3K27ac histone modifications at the SEs exceed those at the typical enhancers by at least an order of magnitude (Whyte et al., 2013). Hence, in the H3K27ac ChIP-Seq binding profiles, we can see that more H3K27ac peaks are enriched in PADI SE in H1975 OR cells compared to the same region in H1975 cells. Interestingly, we found that PADI1, PADI2, and PADI3 expression were inhibited by JQ1, a BETs inhibitor preferentially inhibits SE signaling. This result is consistent with the previous report that the transcription of SEs-associated genes is highly sensitive to bromodomain inhibitors (Lovén et al., 2013). This result further confirms that PADI1, PADI2, and PADI3 genes are driven by SE.

PADI is a transcriptional regulatory protein that affects gene expression. Five PADI family members are identified in the human body, including PADI1-4 and PADI6 (Rus’d et al., 1999; Ishigami et al., 2002; Chavanas et al., 2004). Histone citrullination is a process of modification of arginine residues of histones, driven by Ca^2+^, catalyzed by PADI, and converted to citrulline (Zhu et al., 2021). We hypothesize that one potential mechanism of OR in NSCLC is that SE drives PADI expression to promote histone citrullination. Histones are the main protein components in chromatin and play a central role in gene regulation. PADI-promoting histone citrullination not only affects the formation of tumors but also is associated with the drug resistance of tumors. In a mouse model of pancreatic cancer cell line HCT-116 xenograft, it was found that a potent PADI inhibitor can enhance the activity of antitumor drugs by inhibiting PADI-mediated histone citrullination (Wang et al., 2017). In multiple myeloma, PADI2 is the most up-regulated transcript in bone marrow mesenchymal stem cells, which may mediate the up-regulation of IL-6 expression by inducing histone citrullination, enabling tumor cells to acquire resistance to anti-tumor drugs (McNee et al., 2017). Our study found that the OR H1975 cell line has high PADI expression. Moreover, the PADI inhibitor has no pronounced killing effect on normal H1975 cells but has a more obvious killing effect on drug-resistant H1975 cells, indicating that PADI is a potential therapeutic target in drug-resistant NSCLC. This result also reminds us that detecting the positive expression of PADI in tumor tissue after the acquired OR is crucial if we want to use it as a target.

Various SEs inhibitors have been developed, including BRD4 inhibitors, histone acetylation inhibitors, and CDK inhibitors, and gene editing technology is also a means in research (Zheng et al., 2020; Li et al., 2021). Targeting complexes of SEs is a promising strategy for drug resistance in cancer therapy. In addition, it is possible to use SEs as prognostic markers to predict disease risk and progression. Integrating a gene transcription signature with a SE profile of patients or healthy individuals can provide valuable insights into disease diagnosis. We highlight the critical role of the pathologic generation of SEs on the onset of OR and discuss targeting SEs and their downstream genes for treatment and as biomarkers for progress evaluation.

To sum up, we explored the potential pathogenic SEs and their driven genes in OR NSCLC and found that SE-driven PADIs may be an essential mechanism of OR NSCLC. This study provides evidence for the possibility that PADIs as novel targets and diagnostic biomarkers for OR NSCLC.

## Data Availability

The datasets presented in this study can be found in online repositories. The names of the repository/repositories and accession number(s) can be found in the article/supplementary material.
